# Biochanin A Promotes Osteogenic but Inhibits Adipogenic Differentiation: Evidence with Primary Adipose-Derived Stem Cells

**DOI:** 10.1155/2013/846039

**Published:** 2013-06-16

**Authors:** Shu-Jem Su, Yao-Tsung Yeh, Shu-Hui Su, Kee-Lung Chang, Huey-Wen Shyu, Kuan-Ming Chen, Hua Yeh

**Affiliations:** ^1^Department of Medical Laboratory Science and Biotechnology, School of Medicine and Health Sciences, FooYin University, 151 Chin-Hsueh Road, Ta-Liao, Kaohsiung 83101, Taiwan; ^2^Institute of Medical Sciences, College of Medicine, Tzu Chi University, Hualien, Taiwan; ^3^Department of Biochemistry, College of Medicine, Kaohsiung Medical University, Kaohsiung 80708, Taiwan

## Abstract

Biochanin A has promising effects on bone formation *in vivo*, although the underlying mechanism remains unclear yet. This study therefore aimed to investigate whether biochanin A regulates osteogenic and adipogenic differentiation using primary adipose-derived stem cells. The effects of biochanin A (at a physiologically relevant concentration of 0.1–1 *μ*M) were assessed *in vitro* using various approaches, including Oil red O staining, Nile red staining, alizarin red S staining, alkaline phosphatase (ALP) activity, flow cytometry, RT-PCR, and western blotting. The results showed that biochanin A significantly suppressed adipocyte differentiation, as demonstrated by the inhibition of cytoplasmic lipid droplet accumulation, along with the inhibition of peroxisome proliferator-activated receptor gamma (PPAR**γ**), lipoprotein lipase (LPL), and leptin and osteopontin (OPN) mRNA expression, in a dose-dependent manner. On the other hand, treatment of cells with 0.3 *μ*M biochanin A increased the mineralization and ALP activity, and stimulated the expression of the osteogenic marker genes ALP and osteocalcin (OCN). Furthermore, biochanin A induced the expression of runt-related transcription factor 2 (Runx2), osteoprotegerin (OPG), and Ras homolog gene family, member A (RhoA) proteins. These observations suggest that biochanin A prevents adipogenesis, enhances osteoblast differentiation in mesenchymal stem cells, and has beneficial regulatory effects in bone formation.

## 1. Introduction

Menopause or age-related osteoporosis is associated with a progressive decrease in bone formation and an increase in adipogenesis in the bone marrow, thus increasing the risk of bone fractures [1, 2]. Bone development and homeostasis is mainly determined by the biological balance between osteogenesis and adipogenesis [3]. Recent studies in osteoporotic patients observed abnormal bone marrow-derived stem cells (BMSCs), wherein the BMSCs were deficient in their ability to differentiate into an osteogenic lineage and displayed increased adipogenic potential [4, 5]. 

Adipose-derived stem cells (ADSCs) and BMSCs have been most extensively evaluated as they offer the most accessible source of MSCs for use in research and clinical applications. They also display similar differentiation potentials. However, ADSCs may have greater advantage than BMSCs in clinical uses due to the following reasons. First, ADSCs are relatively abundant and easy to isolate. Second, ADSCs have been shown to have multilineage potential capable of differentiating into adipocyte, chondrocyte, and osteoblast, and they share similar phenotypic and functional characteristics with the BMSCs [6, 7]. Third, MSCs extracted from adipose tissue are less invasive and less expensive than those extracted from bone marrow [8]. Fourth, ADSCs have a significantly shorter doubling time [9]. Fifth, few or no ethical issue is involved in using ADSCs for research and medical purposes. Collectively, these intrinsic characteristics and advantages make ADSCs an ideal stem cell source for cell-based tissue engineering and/or therapies [7, 10].

Additionally, ADSCs were used in the regenerative treatment of traumatic calvarial bone defects in humans [11]. ADSCs can differentiate into multiple mesenchymal lineages, and they are a unique model to improve our understanding of the early differentiation events.

It was reported that inhibiting marrow adipogenesis could help to either prevent or treat osteopenic disorders [12]. Therefore, more functional bone cells can be generated by the inhibition of marrow adipogenesis, with a concomitant increase in osteogenesis; this could either prevent further increases in adipocyte formation or divert existing adipocytes to become more osteoblastic, resulting in increased functional bone cells [13].

The differentiation of adipocytes requires activation by several adipogenesis-related genes, including peroxisome proliferator-activated receptor gamma (PPAR*γ*) and the PPAR*γ*-target gene lipoprotein lipase (LPL) [14]. PPAR*γ* is a key transcription factor that is involved in lipid metabolism and adipocyte differentiation [15]. The knockdown of Osteopontin (OPN), a positive regulator of adipogenesis and a negative regulator of osteoblastic differentiation, enhances osteogenic differentiation and inhibits adipogenic differentiation potential of BMSCs *in vitro* [16]. Remarkably, leptin mRNA increases during differentiation, with the highest levels observed at the end of adipogenic differentiation [14].

 Cytokines released from preadipocytes and other cell types function as the initiators of adipogenesis or osteogenesis. Previous studies have shown that IL-6 and TNF*α* are expressed and upregulated in adipose tissues of obese subjects [17, 18], and they may augment adipocyte differentiation [19]. In addition, differentiated cells show key features of adipocytes such as expression of specific molecular markers and accumulation of lipid droplets in the cytoplasm [20].

The differentiation of osteoblasts requires a distinct series of osteogenesis-related factors, including alkaline phosphatase (ALP), osteocalcin (OCN), osteoprotegerin (OPG), and runt-related transcription factor 2 (Runx2) [21, 22]. During the early stages of osteoblast differentiation, Runx2 induces the differentiation of multipotent mesenchymal cells into immature osteoblasts and triggers the expression of major bone matrix genes, including OCN, ALP, and others [23]. Additionally, Runx2 expression in mesenchymal cells can inhibit differentiation of MSCs into adipocytes by blocking PPAR*γ* activity [24]. Activation of NAD-dependent deacetylase sirtuin-1 (Sirt1) decreases adipocyte formation during osteoblast differentiation of MSCs [25]; however, expressing the dominant-negative Ras homolog gene family, member A (RhoA), committed MSCs to become adipocytes, while the constitutively active RhoA promoted osteogenesis [26].

Nutritional and pharmacological factors such as isoflavones may be an important tool for preventing bone loss associated with aging or menopause [27–29]. Their chemical structure is similar to that of estrogen and enables them to bind the estrogen receptors (either as agonists or antagonists); thus isoflavones might be an alternative to hormone replacement therapy [30, 31], and they are used in phytomedicine to treat menopausal symptoms and osteoporosis. Our previous study had demonstrated that the dietary intake of soy isoflavone extract could prevent bone loss in ovariectomized (OVX) rats, an animal model of postmenopausal osteoporosis [32]. Biochanin A (5,7-dihydroxy-4′-methoxy-isoflavone), a naturally occurring isoflavone that is most commonly found in legumes, especially in red clover (*Trifolium pratense*), is marketed for the treatment of postmenopausal symptoms including hot flashes and osteoporosis [33, 34]. Furthermore, our recent study has established that biochanin A can effectively prevent the OVX-induced increase in bone loss and bone turnover, possibly by increasing osteoblastic activities and decreasing osteoclastic activities [35]. However, little is known about the effect of biochanin A on the changes in differentiation of osteoblasts and adipocytes. In addition, plasma biochanin A concentrations ≤1 *μ*M can be attained with a daily oral intake of 5–50 mg/kg of body weight in rats [36]. Indeed, the maximum plasma concentration of any isoflavone rarely exceeds 1 *μ*M following dietary intake [37]. Thus, in the present study, to elucidate whether biochanin A (at a physiologically relevant concentration of 0.1–1 *μ*M) can directly modulate the differentiation of ADSCs, we examined the effect of biochanin A on adipogenic and osteogenic differentiation and explored the possible mechanism. 

## 2. Materials and Methods

### 2.1. Materials

Biochanin A, *β*-glycerophosphate (*β*GP), ascorbic acid, 3-isobutyl-1-methylxanthine, dexamethasone, insulin, and alizarin red S were obtained from Sigma (MO, USA). Dulbecco's modified Eagle's medium (DMEM), DMEM-F12, penicillin, and streptomycin were obtained from Gibco (NY, USA). Collagenase type II was obtained from Worthington (NJ, USA).

### 2.2. Preparation and Culture of Primary Rat Adipose-Derived Stem Cells (ADSCs)

Primary rat ADSCs from rat adipose tissue were isolated and cultured as previously described [38], with minor modifications. The fatty tissue around the kidneys and testicles of 4-week-old Sprague-Dawley rats was separated. After removal of visible blood vessels, the tissue was finely minced with scissors and digested with collagenase type I (0.15% w/v) (compared to [38] which used 0.1% w/v) in Krebs-Ringer bicarbonate-HEPES buffer (0.11 g/L CaCl_2_, 0.25 g/L MgSO_4_, 0.84 g/L NaHCO_3_, 7 g/L NaCl, 0.55 g/L KH_2_PO_4_, 7.15 g/L HEPES, 10 g/L BSA, and 1 mL of 200 *μ*M adenosine; pH 7.4) (the content used in [38] did not add HEPES) for 60 min at 37°C with shaking. The floating adipocytes were separated by centrifugation at 1200 rpm for 5 min. ADSCs (at a density of 1 × 10^6^) were plated in tissue culture flasks in DMEM (in [38], they used DMEM-F10) supplemented with 10% FBS, 100 U/mL penicillin, and 0.1 mg/mL streptomycin, at 37°C in a humidified atmosphere containing 5% CO_2_. The culture medium was changed every 3 days, and the primary cells were passaged twice before being used for differentiation assays. The cell surface CD29, CD44, and CD90 protein markers of ADSCs were positively expressed and analyzed by flow cytometry at Passage 3 (P3), and the following criteria were used to characterize the ADSCs. The P3 cells passaged at a density of 4 × 10^4^/cm^2^ were used for the experiments.

### 2.3. Induction of Adipogenic Differentiation

For adipocyte differentiation, ADSCs were cultured in adipogenic differentiation medium containing DMEM supplemented with 0.25 mM 3-isobutyl-1-methylxanthine, 1 mM dexamethasone, and 1 *μ*mol/L insulin (MDI medium), in the presence or absence of biochanin A (0.1–1 *μ*M) for 12 days. Media and treatments were changed every 3 days.

### 2.4. Lipid Droplet Staining with Oil Red O

Cell monolayers were rinsed twice with PBS and fixed with 10% formaldehyde for 30 min at room temperature. After washing with distilled water 2 times, cells were stained with filtered 3 mg/mL Oil red O dissolved in 60% isopropanol for 10 min at room temperature. The plates were placed on an orbital shaker for 1 h and then rinsed with 70% ethanol to remove the background Oil red O stain [39, 40].

### 2.5. Quantification of Adipocyte Number by Flow Cytometry

Adipocytes were quantified after adipogenic differentiation using lipophilic Nile red flow cytometry analysis [41–44]. In brief, cells were carefully trypsinized, centrifuged for 10 min at 200 ×g at 4°C, washed twice with PBS, and fixed with 10% formaldehyde at 4°C. For flow cytometry, the cells were stained with 1 *μ*g/mL Nile red for 30 min at 37°C. The samples were then analyzed with a FACScan flow cytometer (Beckman Coulter), and Nile red fluorescence was measured on the FL2 emission channel using a 575 nm band-pass filter. Data analysis was performed using the WinMDI 2.9 software. 

### 2.6. Total RNA Extraction and Reverse Transcriptase-Polymerase Chain Reaction (RT-PCR)

Total RNA was extracted from the left femur using REzol reagent (Protech, Taiwan). Reverse transcriptase-polymerase chain reaction (RT-PCR) was performed as described previously [23]. To synthesize complementary DNA (cDNA), 2 *μ*g of RNA was resuspended in 12.5 *μ*L of diethylpyrocarbonate-treated water, 1 *μ*L of oligo (dT) primer was added, and the mixture was annealed for 5 min at 70°C. The sample was then cooled to 4°C for 2 min before addition of 4 *μ*L of 5× reaction buffer (50 mM Tris-HCl, 75 mM KCl, and 3 mM MgCl_2_; pH 8.3), 0.5 *μ*L of RNase inhibitor, 1 *μ*L of 10 mM dNTP, and 1 *μ*L of Maloney's murine leukemia virus reverse transcriptase (Promega, Lyon, France). The reaction mixture was heated for 60 min at 37°C to synthesize the cDNA, and it was then stopped by denaturing the enzyme at 94°C for 5 min. cDNA was amplified by PCR to generate the genes listed as follows: PPAR*γ*2 (NM_013124): forward: 5′-caggcttgctgaacgtgaag-3′, reverse: 5′-acgtgctctgtgacaatctgc-3′ (177 bp); OPN (M99252): forward: 5′-cctgcaccaccaactgctta-3′, reverse: 5′-ggccatccacagtcttctgag-3′ (208 bp); LPL (NM_012598): forward: 5′-actgccacttcaaccacagc-3′, reverse: 5′-gtatcgggcccagcaacatt-3′ (225 bp); leptin (NM_013076): forward: 5′-cacaca cgcagtcggtatcc-3′, reverse: 5′-cacgttttgggaaggcaagc-3′ (146 bp); IL-6 (NM_012589): forward: 5′-ccagccagttgccttcttg-3′, reverse: 5′-gagagcattggaagttgggg-3′ (496 bp); TNF*α* (X66539) forward: 5′-tgagcacagaaagcatgatcc-3′, reverse: 5′-gctcttgatggcggagagg-3′ (530 bp); ALP (NM_013059): forward: 5′-tggacggtgaacgggagaac-3′, reverse: 5′-cagagctggcccaggcaca-3′ (238 bp); and osteocalcin (NM_013414): forward: 5′-tgaggaccctctctctgctc-3′, reverse: 5′-accaccttactgccctcctg-3′ (130 bp). GAPDH (NM_017008): forward: 5′-cctgcaccaccaactgctta-3′, reverse: 5′-ggccatccacagtcttctgag-3′ (140 bp), was amplified as a housekeeping gene. PCR amplification was performed for 30 cycles at 94°C for 1 min, 62°C for 1 min, and 72°C for 1 min, followed by 7 min at 72°C. The amplified PCR products were separated by gel electrophoresis in a 2% agarose gel visualized with ethidium bromide, and with the intensity of each band calculated by densitometric analysis and the results expressed as a percentage of the density of the corresponding GAPDH band.

### 2.7. Induction of Osteogenic Differentiation

For osteogenic differentiation, ADSCs were cultured in an osteogenic medium (OM) consisting of DMEM-F12 medium supplemented with 50 *μ*g/mL ascorbic acid, 10 nM dexamethasone, and 3 mM *β*-glycerophosphate, in the absence or presence of biochanin A (0.1–1 *μ*M) for 12 days. Media and treatments were replaced every 3 days. After treatment, the cells were collected for analysis of osteogenic-specific markers by RT-PCR and western blot. The cells were also analyzed for ALP activity. After 12 days, the mineral deposition in osteogenic differentiation assays was visualized by alizarin red S staining.

### 2.8. Staining for Mineralization

After 12 days of incubation with OM supplemented with different concentrations of biochanin A, the mineral deposition in the extracellular matrix was visualized by staining with alizarin red S. Briefly, the cells were washed with PBS and fixed with 70% ethanol for 1 hour at −20°C. The cells were then rinsed in distilled water, stained with 40 mM alizarin red S (Sigma-Aldrich) at pH 4.2 with rotation for 10 min at room temperature, and subsequently washed with distilled water [45]. The stained areas were measured using a semiautomatic image-analyzing program (Mac Scope, Mitani, Fukui, Japan).

### 2.9. Assessment of Cellular Alkaline Phosphatase (ALP) Activity

After 12 days of incubation in OM containing different concentrations of biochanin A, ADSCs were rinsed twice with 1×PBS, suspended, and then lysed in 0.1 mL of 2 mM Tris-HCl containing 1% Triton-X-100 solution. The collected cells were subsequently homogenized by sonication and centrifuged at 4°C for 20 min at 12,000 rpm, and the supernatants were assayed for ALP activity. ALP activity was measured using an ALP assay kit (alkaline phosphatase yellow liquid substrate system for ELISA; Sigma-Aldrich Inc.); this involved measuring the p-nitrophenol produced after 30 min. The absorbance of the reaction solution was measured at 405 nm using a microplate reader, and it was then converted to a nitrophenol concentration by comparison with a standard dilution series of p-nitrophenol. One unit of activity (1 U) corresponds to the production of 1 g of p-nitrophenol. All of the data were normalized for total protein content by dividing the amount of ALP of cells. Total protein was then measured using the Bio-Rad D*c* protein assay reagent. 

### 2.10. Western Blotting

Cytosolic extracts were prepared from cells, and the protein in the supernatant was quantified using a protein assay kit (Bio-Rad Laboratories, Hercules, CA, USA). A sample (60 *μ*g) was electrophoresed on a 12% SDS-polyacrylamide gel, and it was subsequently transferred onto a nitrocellulose membrane. After blocking, the membrane was probed with Runx2, OPG, RhoA, Sirt 1, and *β*-actin antibodies (Santa Cruz Biotechnology), followed by the appropriate horseradish peroxidase-labeled secondary antibody (PharMingen, San Diego, CA, USA). The bound antibody was quantified by chemiluminescence detection (PerkinElmer Life Sciences Inc.). *β*-actin was used as an internal control. The amount of the test protein, expressed as arbitrary densitometric units, was normalized to *β*-actin, and then the density of the band was expressed as the relative density compared to that for untreated cells (control), which was set at 100%.

### 2.11. Statistical Analysis

All quantitative data were expressed as mean ± SD. Statistical analysis was performed by SigmaPlot 7.0, using a paired *t*-test. Results with *P* < 0.05 were considered statistically significant.

## 3. Results

### 3.1. Dose-Response Effects of Biochanin A on Adipogenic Differentiation of ADSCs

Results from flow cytometric analysis indicated that ADSCs at Passage 3 used in this study were immunopositive (>95%) for CD29, CD44, and CD90. Furthermore, biochanin A (0.1–1 *μ*M) did not affect ADSCs cell viability (*P* > 0.05), as determined using trypan blue dye exclusion and crystal violet staining.

**Figure 1:**
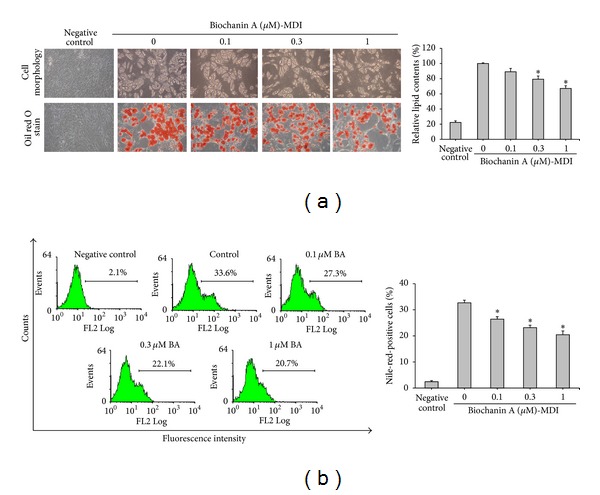
Biochanin A (BA) inhibited the adipogenic differentiation of ADSCs. ADSCs were treated for 12 days with a basic medium (negative control) or an adipogenic medium (MDI) in the presence of 0.1–1 *μ*M biochanin A. (a) After 12 days of incubation, the cells were fixed and adipogenic differentiation was determined by the Oil red O staining of lipid droplets. Morphological changes in cells were observed using a light microscope (upper and lower panel, original magnification: 200x). Lipid droplets were quantified using Oil red O dissolved in isopropanol and by determining absorbance at 490 nm. (b) After 12 days of incubation, the cells were harvested, fixed, and stained with Nile red solution. Percentage of Nile-red-stained cells in the total population of each sample was quantified with FACScan flow cytometry. All results are expressed as the mean ± SD of three independent experiments. *P < 0.05 compared with the control.

Adipogenic differentiation involves dramatic changes in the cellular morphology and gene expression [46]. Morphological observations include the presence of lipid droplets in the adipocyte cytoplasm, which show a positive stain with Oil red O. Previous studies have used Nile red flow cytometry for assessing adipocyte cell numbers [41–44]. To determine the effects of biochanin A on adipogenic differentiation of ADSCs, the cells were treated with different concentrations of biochanin A (0.1, 0.3, and 1 *μ*M) for 12 days, and lipid accumulation was observed using the Oil red O staining (Figure 1). Minimal lipid accumulation was observed in cells cultured in the basic medium (negative control; Figure 1), while ADSCs cultured in the adipogenic medium (MDI medium) for 12 days showed a significant increase in cellular lipid accumulation of up to 75% compared with the negative control. Biochanin A decreased the lipid droplet accumulation in a dose-dependent manner (Figure 1(a)). Flow cytometry and Nile red staining were used to calculate the percentage of cells that were positively stained as adipocytes. Following culture in the adipogenic medium for 12 days, 33.6% of the cells were found to be adipocytes; treatment with 0.1, 0.3, or 1 *μ*M biochanin A reduced the number of adipocytes to 27.3%, 22.1%, and 20.7%, respectively (Figure 1(b)).

Similarly, biochanin A also significantly and dose dependently inhibited lipid accumulation and adipocyte formation in M2-10B4 mouse bone marrow stromal cells (data not shown).

### 3.2. Effects of Biochanin A on the Expression of Adipogenesis-Related Factors and Cytokines

**Figure 2:**
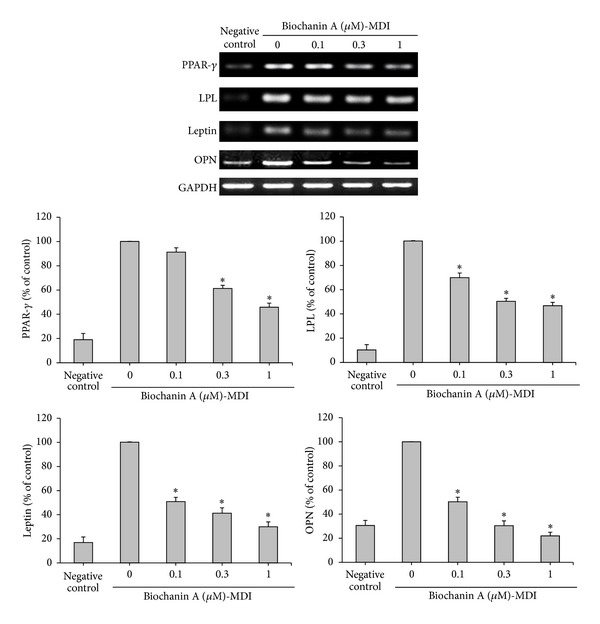
Biochanin A inhibited the expression of adipogenesis-related factors in ADSCs. ADSCs were treated for 12 days with a basic medium (negative control) or an adipogenic medium (MDI) in the presence of 0.1–1 *μ*M biochanin A. After 12 days of incubation, the expression of PPAR*γ*, LPL, leptin, and OPN mRNA was measured by RT-PCR. GAPDH expression was used for normalization. All results are expressed as the mean ± SD of three independent experiments. *P < 0.05 compared with the control.

To investigate the mechanism of the inhibitory effects of biochanin A on adipogenesis, RT-PCR was performed to determine the expression of adipogenesis-related factors PPAR*γ*, LPL, leptin, and OPN genes. Biochanin A decreased the expression of PPAR*γ* and the PPAR*γ*-target gene LPL (*P* < 0.05). Additionally, biochanin A also markedly suppressed leptin, and the OPN gene in a dose-dependent manner (*P* < 0.05). These findings suggest that biochanin A effectively inhibited adipogenesis signaling in ADSCs (Figure 2). 

**Figure 3:**
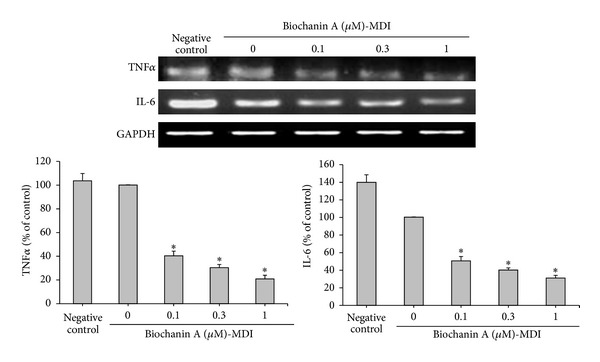
Biochanin A inhibited the expression of adipogenesis-related cytokines in ADSCs. ADSCs were treated for 12 days with a basic medium (negative control) or an adipogenic medium (MDI) in the presence of 0.1–1 *μ*M biochanin A. After 12 days of incubation, expression of TNF*α* and IL-6 mRNA was measured by RT-PCR. GAPDH expression was used for normalization. All results are expressed as the mean ± SD of three independent experiments. *P < 0.05 compared with the control.

Recent insights into the metabolic and immunological functions of preadipocytes showed that these cells are potent producers of proinflammatory cytokines such as TNF*α* and IL-6 [47], and previous study has shown that ADSCs secrete IL-6 and TNF*α* [6]. In the present study, ADSCs continued to express TNF*α* and IL-6 in the basic medium (negative control group) (Figure 3); however, when cultured in the adipogenic medium (control group), TNF*α* and IL-6 levels were lower than those in the control group (Figure 3). Moreover, biochanin A remarkably inhibited TNF*α* and IL-6 expressions in a dose-dependent manner (Figure 3) (*P* < 0.05).

### 3.3. Effects of Biochanin A on Osteogenic Differentiation of ADSCs

**Figure 4:**
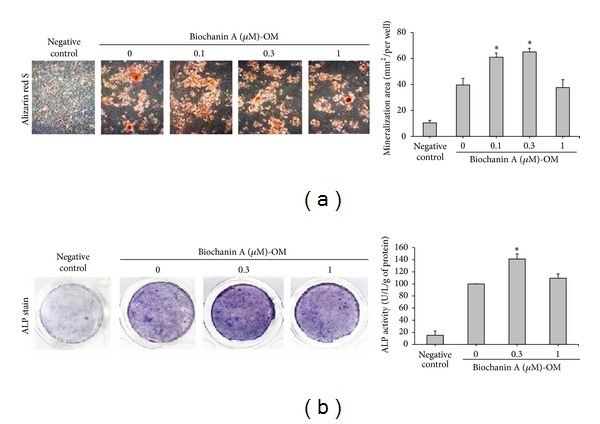
Biochanin A enhanced the osteogenic differentiation in ADSCs. ADSCs were treated for 12 days with a basic medium (negative control) or an osteogenic medium (OM) in the presence of 0.1–1 *μ*M biochanin A. (a) Osteogenic differentiation was determined by alizarin red S staining of calcium deposits (mineralization) within the cell monolayer. (b) ALP activity was determined using the ALP assay kit according to the manufacturer's protocol. All results are expressed as the mean ± SD of three independent experiments. *P < 0.05 compared with the control.

**Figure 5:**
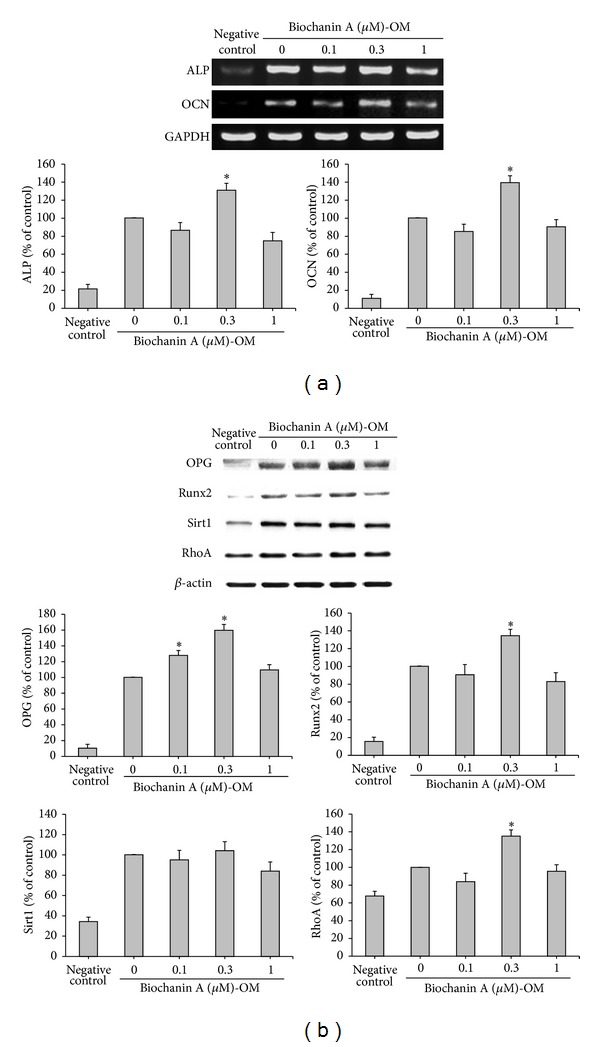
Biochanin A enhanced the expression levels of genes and proteins that regulate osteogenic differentiation in ADSCs. ADSCs were treated for 12 days with a basic medium (negative control) or an osteogenic medium (OM) in the presence of 0.1–1 *μ*M biochanin A. (a) After incubation, expression of ALP, OCN, and GAPDH mRNA was measured by RT-PCR. Expression of ALP and OCN genes was normalized to that of GAPDH. (b) After incubation, total proteins were isolated and analyzed for the expression of OPG, Runx2, Sirt1, and RhoA proteins by western blot analysis. *β*-actin was used as the internal control. All results are expressed as the mean ± SD of three independent experiments. *P < 0.05 compared with the control.

To address whether or not biochanin A affects osteogenic differentiation in ADSCs, the cells grown in OM (consisting of basic medium plus 50 *μ*g/mL ascorbic acid and 3 mM *β*-glycerophosphate) were treated with various concentrations of biochanin A for 12 days. Only a small percentage of ADSCs grown in a basic medium (consisting of DMEM-F12 and 10% FBS only) differentiated into osteoblasts (Figure 4, negative control). The OM significantly induced calcium deposition and ALP activity in the ADSC monolayer, as determined by alizarin red S staining and the ALP assay kit, respectively (Figure 4, control), and biochanin A further stimulated these processes, with a maximum effect observed at 0.3 *μ*M (Figure 4). Additionally, analysis of the mRNA by RT-PCR showed that, following osteoblast differentiation and treatment with 0.1 *μ*M biochanin A, expression of ALP and OCN (both osteogenic differentiation marker genes) was not significantly different when compared to the control group (*P* > 0.05) (Figure 5(a)). Notably, biochanin A at 0.3 *μ*M could significantly upregulate the expression of the osteoblast marker genes ALP and OCN (*P* < 0.05) (Figure 5(a)). 

### 3.4. Effects of Biochanin A on the Expression of Proteins That Regulate Osteogenesis

To further investigate the influence of biochanin A on the expression of osteogenic proteins, the present study evaluated the expression of OPG, Runx2, Sirt1, and RhoA by western blotting. These proteins are upregulated in osteogenesis (Figure 5(b), control), and treatment with 0.3 *μ*M biochanin A demonstrated significantly enhanced OPG, Runx2, and RhoA protein expression in OM, compared with control cells (*P* < 0.05) (Figure 5(b)). The 0.3 *μ*M dose of biochanin A exerted the greatest biological activity (Figures 4 and 5), while other doses did not have significantly different effects from the control cells. Biochanin A did not induce a significant change in Sirt1 protein expression compared with the control cells (*P* > 0.05). Taken together, these data demonstrated that biochanin A has biphasic effects on the regulation of adipogenesis and osteogenesis.

## 4. Discussion

ADSCs have been shown to possess multilineage potential, and are capable of differentiating into adipogenic, osteogenic, myogenic, and chondrogenic cells that display functional characteristics comparable to those of stem cells obtained from the bone marrow [7, 48]. Additionally, ADSCs are an ideal source of stem cells for cell-based tissue engineering and therapies [7]. A recent study showed that aging impedes the potential of ADSCs to improve osteoporosis by diminishing the osteogenic signaling, and it also showed that ADSCs could be used as a potential cell-based therapy for osteoporosis [49]. 

Increased bone resorption and decreased bone formation play key roles in the occurrence and development of osteoporosis. However, apart from excessive bone resorption, an increase in adipose tissue was frequently observed in the bone marrow stroma of osteoporotic patients [50], which implied the importance of adipogenesis in bone loss [51]. Our previous study had established that biochanin A inhibits osteoclast formation and decreases osteoclast bone resorption *in vitro*; additionally, it inhibits the production of bone resorption markers in an OVX animal model [35]. Furthermore, biochanin A induced preosteoblasts to differentiate into osteoblasts, increased osteoblast mineralization, and effectively prevented the OVX-induced increase in bone loss [35]. However, the role of biochanin A in regulating the differentiation of mesenchymal stem cells into adipocyte or osteoblast lineages has not been yet reported. 

The balance between osteogenic differentiation and adipogenic differentiation in BMSCs plays an important role in osteoporosis [52, 53]. Research has shown that medullary adipocytes are secretory cells from the bone marrow stroma that may influence osteogenesis by impairing osteoblast proliferation, differentiation, and mineralization, and by promoting osteoclast formation and activation [54, 55]. Therefore, inhibition of the differentiation of marrow adipocytes coupled with increased osteogenesis may provide a strategy for the treatment of osteoporosis. 

An important finding of the current study was that biochanin A promotes osteogenesis and inhibits adipogenesis in ADSCs, as evidenced by a decrease in adipocyte numbers, lipid droplets, and the expression of adipogenic marker genes, in addition to an increase in osteoblast numbers, ALP activity, mineralization, and the expression of osteogenic markers. In the present study, we also observed that the dose range of biochanin A used (0.1–1 *μ*M) did not influence the viability of ADSCs, as determined by the exclusion of trypan blue and crystal violet staining. 

Expression of PPAR*γ* and its target gene LPL, which regulates adipogenic differentiation [56], was markedly decreased in ADSCs treated with biochanin A (Figure 2). A recent study reported that leptin-deficient and leptin receptor-deficient mice had increased bone formation [57]. Leptin may decrease bone formation through the sympathetic nervous system [58]. Notably, OPN has previously been shown to promote adipogenesis [16]; as shown in Figure 2, ADSCs cultured in the adipogenic medium (MDI medium) for 12 days showed significantly increased leptin and OPN expression, which was inhibited by biochanin A in a dose-dependent manner. 

In addition, TNF-*α* and IL-6 clearly inhibited the adipocyte differentiation of MSCs [59]. Our results show that TNF*α* and IL-6 are expressed and upregulated in ADSCs (Figure 3, negative control), but IL-6 was markedly reduced, and TNF*α* was only slightly reduced in ADSCs during adipogenesis (Figure 3, control). Treatment with different doses of biochanin A revealed that TNF*α* and IL-6 mRNA expression level was significantly reduced in a dose-dependent manner (Figure 3). The result is consistent with our previous finding that biochanin A suppressed OVX-induced increase in the serum levels of TNF*α* [35]. This corroborates our observation that biochanin A inhibited adipocyte differentiation and reduced the mRNA expression of adipogenesis-related genes PPAR*γ*, LPL, leptin, OPN, TNF*α*, and IL-6 (Figures 2 and 3). 

Furthermore, we have shown that biochanin A increased ALP activity and mineralization, which confirms that biochanin A stimulates osteoblast differentiation. Our study also found that biochanin A enhanced the transcription of genes encoding the osteoblast differentiation markers ALP and OCN. Notably, biochanin A stimulated osteogenesis with a maximum effect at 0.3 *μ*M, a physiologically relevant concentration that can be achieved by supplementing the nutritional intake. 

Two key transcription factors, Runx2 and PPAR*γ*, drive MSCs to differentiate into either osteoblasts or adipocytes, respectively [60, 61]. Runx2, a bone-specific transcription factor, is a key regulator of osteoblast differentiation. However, the expression of PPAR*γ* in mesenchymal cells inhibited their differentiation into osteoblasts by blocking the Runx2 activity [62]. Biochanin A induced the differentiation of MSCs into osteoblasts mainly *via* the augmentation of Runx2 expression (Figure 5(b)). The activated RhoA is sufficient for Runx2 upregulation [63], and activation of Sirt1 decreases adipocyte formation during osteoblast differentiation of mesenchymal stem cells [41]. ADSCs undergoing osteogenic commitment can differentiate into osteoblasts, with a concomitant upregulation of Runx2 and RhoA protein expression in osteogenesis (Figure 5(b)). At the 0.3 *μ*M dose, biochanin A significantly increased osteogenesis and inhibited adipogenesis in ADSCs (Figures 1–5) (*P* < 0.05) and greatly enhanced the expression of Runx2 and RhoA while inhibiting that of PPAR*γ* (Figures 2 and 5(b)) (*P* < 0.05), but it did not change Sirt1 protein expression (Figure 5(b)). On the other hand, the strong effects of biochanin A on osteogenic protein expression are seen on OPG. OPG is a secreted decoy receptor for RANKL, which is expressed by stromal/osteoblast cells, and RANKL is essential for the maturation and activity of osteoclasts [64]. OPG inhibits osteoclastogenesis by preventing the interaction between RANKL-RANK [65]. OPG-deficient mice exhibit severe trabecular and cortical bone porosity, with enhanced osteoclastic bone resorption [66]. Thus, the balance between the expression of OPG and RANKL equalizes bone formation and resorption during bone remodeling. Our previous experiments indicate that biochanin A exerts a positive effect on the bones, not only by increasing osteoblast formation and osteoblastic activities, but also by suppressing osteoclast formation and bone resorption *in vitro* and *in vivo*. Furthermore, biochanin A decreased the ratio of RANKL/OPG mRNA expression in the femur of OVX rats [35]. Therefore, our studies clearly demonstrate that the stimulating effect of biochanin A on bone formation *via* increased osteogenic differentiation and inhibited adipogenic and osteoclastic differentiation, resulting in increase in bone mass.

Because of its structure similarity to estradiol, biochanin A may act as potential replacement for estrogen deficiency and may therefore be useful in prevention and treatment of postmenopausal osteoporosis. However, biochanin A has different actions compared with estrogen based on selective estrogen receptor modulator activity; it is preferentially bound to estrogen receptor *β* (found in the bone, vasculature, and heart) rather than estrogen receptor *α* (found in the breast, ovaries, uterus, and adrenal glands) [67].

Since stimulation of osteogenesis and concomitant inhibition of adipogenesis may explain the fact that estrogen deficiency decreases bone mass and increases adipose tissue, as seen in postmenopausal women, supplementation of red clover isoflavones has positive effects on bone density [27, 68]. Biochanin A exerts beneficial effects on the bone, which is also well documented [35]. Furthermore, the present study provides corroborative evidence for the first time that biochanin A inversely regulates osteogenic and adipogenic commitment of ADSCs. Biochanin A promotes bone formation by driving the differentiation of adipose-derived stem cells into osteoblasts rather than adipocytes, as indicated by the downregulation of PPAR*γ*, LPL, leptin, OPN, TNF*α*, and IL-6 expression, in consort with the upregulation of ALP, OCN, OPG, Runx2, and RhoA expression.

The safety of supplementary biochanin A or red clover isoflavones had been studied; supplement 40 mg red clover isoflavones (Promensil, Novogen) (contain 60% biochanin A) did not adversely affect breast density, cardiovascular, or endometrial status in women [69]. It is noted that biochanin A is the main content of red clover isoflavones. The results suggest that biochanin A-containing dietary supplements are safe and well tolerated in human body. Currently 40–50 mg of red clover isoflavones are recommended as daily dose [70]. A supplementary intake of biochanin A may provide an alternative strategy in the prevention of osteoporosis.

## Supplementary Material

Biochanin A (BA) inhibited the adipogenic differentiation of M2-10B4. M2-10B4 were treated for 7 days with a basic medium (negative control) or an adipogenic medium (MDI) in the presence of 0.1–1 M biochanin A. (Left) After 7 days of incubation, the cells were fixed and adipogenic differentiation was determined by the Oil red O staining of lipid droplets. (Right) After 7 days of incubation, the cells were harvested, fixed, and stained with Nile red solution. Percentage of Nile-red-stained cells in the total population of each sample was quantified with FACScan flow cytometry. All results are expressed as the mean ± SD of three independent experiments. *P* < 0.05 compared with the control.
