# Supplementary figures and images for: Biochanin A Promotes Osteogenic but Inhibits Adipogenic Differentiation: Evidence with Primary Adipose-Derived Stem Cells
Source: Evid Based Complement Alternat Med. 2013 Jun 16;2013:846039. doi: 10.1155/2013/846039 (PMC3697292; doi:10.1155/2013/846039)

Supplementary data

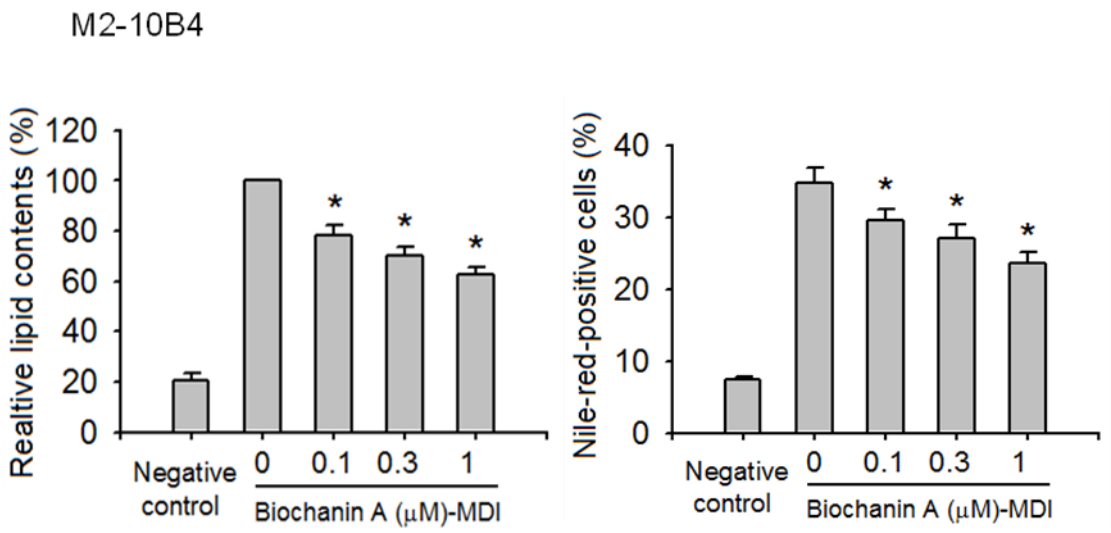

Supplement: Supplementary file 1 — Biochanin A (BA) inhibited the adipogenic differentiation of M2-10B4. M2-10B4 were treated for 7 days with a basic medium (negative control) or an adipogenic medium (MDI) in the presence of 0.1–1 M biochanin A. (Left) After 7 days of incubation, the cells were fixed and adipogenic differentiation was determined by the Oil red O staining of lipid droplets. (Right) After 7 days of incubation, the cells were harvested, fixed, and stained with Nile red solution. Percentage of Nile-red-stained cells in the total population of each sample was quantified with FACScan flow cytometry. All results are expressed as the mean ± SD of three independent experiments. P < 0.05 compared with the control. [file 846039.f1.pdf]
